# PVA and CS cross-linking combined with *in situ* chimeric SiO_2_ nanoparticle adhesion to enhance the hydrophilicity and antibacterial properties of PTFE flat membranes

**DOI:** 10.1039/c9ra02396h

**Published:** 2019-06-18

**Authors:** Chengcai Li, Hang Zhang, Feng Wang, Hailin Zhu, Yuhai Guo, Meiyu Chen

**Affiliations:** Zhejiang Provincial Key Laboratory of Fiber Materials and Manufacturing Technology, Zhejiang Sci-Tech University Hangzhou 310018 China zhhailin@163.com; Zhejiang Kertice Hi-Tech Fluor-Material Co., LTD Huzhou 313000 People's Republic of China; School of Textile Science and Engineering, Xi'an Polytechnic University Xi'an 710048 China yuanshijidi@163.com

## Abstract

Herein, a new hydrophilic and antibacterial polytetrafluoroethylene (PTFE) flat MF membrane was fabricated *via* a low-cost and simple preparation method in which chitosan (CS) was crosslinked with poly(vinyl alcohol) (PVA) using epichlorohydrin (ECH) as a cross-linker followed by *in situ* chimeric SiO_2_ nanoparticle adhesion. The surface of the modified membrane had decreased C and F contents, and a large number of hydrophilic groups appeared. The treated membrane had good hydrophilicity and antibacterial properties. Moreover, the PTFE-modified membrane had high separation efficiency and antifouling property for oil-in-water emulsions. Finally, the hydrophilic stability of the PTFE membrane was studied by subjecting it to continuous water rinsing and soaking in solutions of different pH values. The present study demonstrates that this modified membrane has potential practical applications in industrial wastewater recovery.

## Abstract 2

Herein, a new hydrophilic and antibacterial PTFE flat MF membrane was fabricated *via* a low-cost and simple preparation method in which CS was crosslinked with PVA using ECH as a cross-linker followed by *in situ* chimeric SiO_2_ nanoparticle adhesion.

## Graphical abstract

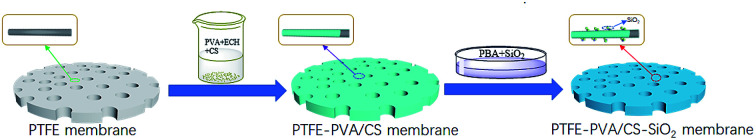

## 1. Introduction

With the increasing seriousness of environmental pollution caused by industrial wastewater and oil spills, research on oil/water separation is receiving significant attention in recent years;^1–4^ however, there are many shortcomings, including low efficiency, high cost, and secondary pollution, of conventional oil–water separation technology;^5–7^ among the oil–water separation techniques, membrane technologies are extensively used in water treatment due to their high space utilization, energy-saving nature, environmental friendliness and cost-effectiveness. Among the organic membranes, the polytetrafluoroethylene (PTFE) MF membrane has many excellent properties such as good thermal stability, high mechanical strength, narrow pore size distribution and high porosity.^8–10^ Due to the symmetric molecular structure of PTFE, the surface tension and friction coefficient are very low; this makes the PTFE membrane exhibit high hydrophobicity characteristics, which greatly limit its application in the field of wastewater treatment.^11,12^ Moreover, superhydrophobic materials tend to be fouled by oil due to their oleophilicity, and the oil–water separation flux of the membrane gradually declines with the increasing cycle times. On the other hand, superhydrophilic membranes show the advantages of antifouling and reusability because they can effectively avoid or reduce external oil fouling by the formation of water barriers between the membranes and the oil phase;^13–16^ in addition, to prevent bacteria from propagating in the pores of the membrane and causing clogging of the membrane pores, the membrane should have antibacterial property. Therefore, the development of hydrophilic and antibacterial PTFE membranes for wastewater treatment has broad application prospects.

In the past few decades, there have been two major methods of improving the hydrophilicity of PTFE membranes; one method involves the destruction of the C–F bond, and the grafting of some hydrophilic groups;^17–21^ the other method involves the application of a layer of hydrophilic coating directly on the membrane surface;^22,23^ however, these two modification methods have some shortcomings. For example, radiation and plasma treatment grafting require complex technology and expensive equipment; moreover, the most important aspect is the grafting uniformity, which cannot be produced on a large scale. Compared with surface grafting methods, the surface coating method is characterized by simple operation and low cost; however, the micropores are often blocked; due to this, the water flux of the modified membrane becomes very low. In addition, few studies have been reported on the antibacterial properties of the PTFE membrane. Therefore, it is essential to develop a hydrophilic and antibacterial PTFE membrane by a low-cost and simple method.

Polyvinyl alcohol (PVA) is a well-known material that is highly soluble in water, non-toxic, biocompatible, hydrophilic, innocuous and non-carcinogenic.^24,25^ PVA, with its abundant hydroxyl groups^26,27^ and good chemical resistance, has been used as a hydrophilic additive; however, to render it stable in an aqueous phase, PVA must be cross-linked by another material (*e.g.*, glutaraldehyde) that can reduce its water solubility. Moreover, for the hydrophilic coating of the composite membrane, polyvinyl alcohol (PVA) is suitable due to its inherent hydrophilicity and good spinnability that make it attractive for preventing oil-fouling. Similarly, chitosan (CS) is prepared by the deacetylation of chitin and has some advantages such as being non-toxic, biodegradable and relatively inexpensive.^28^ In addition, CS and CS derivatives are often used as antibacterial materials.^29–32^ However, the modified membrane does not achieve sufficient hydrophilicity *via* pure CS treatment; this leads to poor anti-fouling properties of the membrane.

The approach of combining CS with other polymers opens a window of research on the alteration or tailoring of the properties of interest. Moreover, the cross-linking of PVA and CS with epichlorohydrin (ECH) not only reduces the water solubility of PVA but also improves the antibacterial properties of the PTFE membrane. The objective of this study was to develop a simple and low-cost facile technique for the fabrication of membranes with significant hydrophilicity, antibacterial activity, and antifouling property.

In this study, we introduced the PVA/CS hydrophilic layer into the fibril surface of the PTFE membrane to improve the hydrophilicity and antibacterial properties of this membrane. Moreover, to better improve the hydrophilicity of the modified PTFE flat membrane, a secondary treatment was conducted on the PVA/CS compound coating. The surface was characterized by Fourier transform infrared (FTIR) spectroscopy and X-ray photoelectron spectroscopy (XPS). The membrane was examined in terms of the water flux and contact angle. In order to further improve the hydrophilic property of modified PTFE membrane, the surface of the modified membranes was treated by adhesion SiO_2_ nanoparticles. Finally, the oil-in-water emulsion separation, the antifouling properties and hydrophilicity stability of the modified PTFE flat membrane were also investigated.

## 2. Experimental

### 2.1 Materials and reagents

**Fig. 1:**
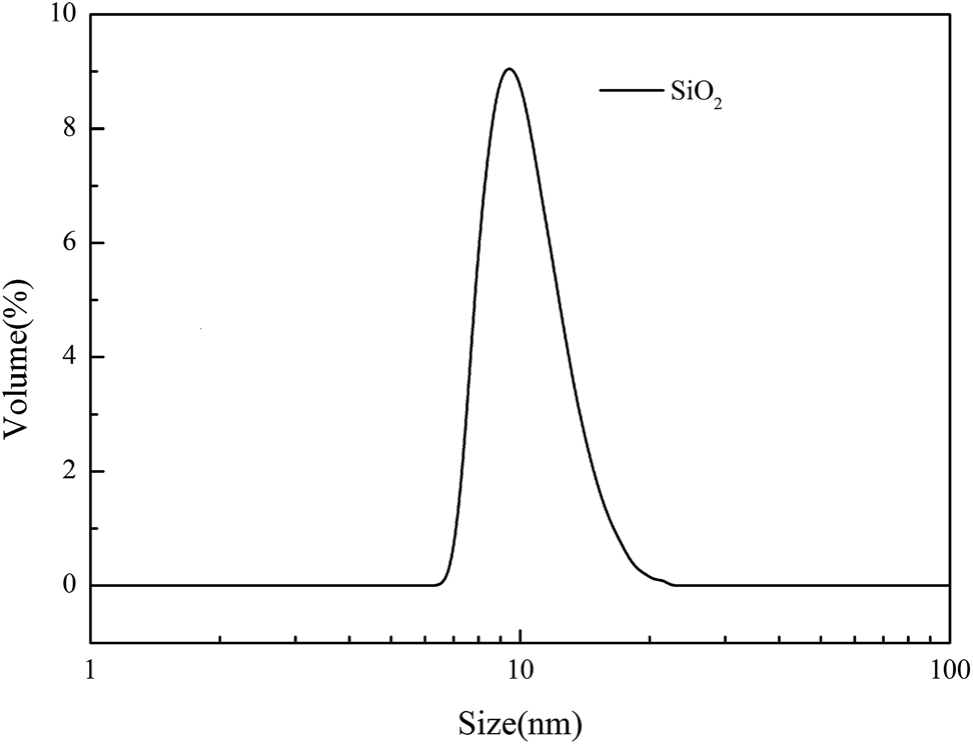
The DLS data for the silica solution.

The PTFE flat MF membrane (mean pore size: 0.2 μm and porosity 80%) was received from Zhejiang Kertice Hi-tech Fluor-material Co. Ltd. PVA (polymerization degree, 1700; hydrolysis degree, 99%) was purchased from Kuraray Co. Ltd. CS (deacetylation degree ≧ 95%; viscosity 100–200 mPa s) was obtained from Shanghai Macklin Biochemical Co. Ltd. Epichlorohydrin (ECH) (99%) was supplied by Tianjin Kermel Chemical Reagent Co. Ltd. KOH (85%) was purchased from Wuxi Zhanwang Chemical Reagent Co. Ltd. The silica solution (the SiO_2_ content is 50%, average size: 15.93 nm, and particle size distribution is shown in Fig. 1) was obtained from Zhejiang Yuda Chemical Co., Ltd. Butyl acrylate (BA) was purchased from Jinan Shijitongda Chemical Co. Ltd. Other reagents, such as sodium dodecyl sulfate (SDS), K_2_S_2_O_8_, acetone, ethanol and Tween-80, were obtained from Hangzhou Gaojing Fine Chemical Industry Co. Ltd. All chemical reagents were used as received without further purification.

### 2.2 Preparation of the PBA/SiO_2_ solution

Mixed solution A: poly(butyl acrylate) (PBA) was prepared by simple emulsion polymerization. SDS (0.1 g) was dissolved in 100 g of deionized water by mechanical stirring at 300 rpm for 10 min; then, 10 g BA was added to the abovementioned solution, and the temperature was adjusted to 80 °C. After this, 0.1 g of K_2_S_2_O_8_ (dissolved in 10 g of deionized water) was added, and the mixture was reacted at 300 rpm and 80 °C for 6 h. The mixed solution B was the silica solution. The PBA/SiO_2_ solution was prepared by mixing the same quantity of A and B at 25 °C.

### 2.3 Preparation of the hydrophilic membrane

The PVA solution (1 wt%) was prepared by polymer dissolution in deionized water under stirring for 2 h at 98 °C. CS was dissolved in 2 wt% acetic acid under stirring at 25 °C for 12 h. The CS and PVA solutions were mixed at certain mass ratio followed by stirring at 25 °C for 1 h to obtain a PVA/CS solution, and then, ECH (6 wt%) was added to this solution mixture. Unless otherwise specified, in the following experiments, the concentrations of CS and PVA were 0.3 wt% and 1 wt%, respectively, and the mixing ratio was 1 : 1.5. The PTFE flat membrane samples were pre-wetted with ethanol for 0.5 h and then transferred into the freshly prepared mixture solution. The KOH solution (50 wt%) was added to the abovementioned mixture solution under stirring, and stirring was continued at 40 °C. After conducting the reaction for a designated time, the modified membranes were drawn out, rinsed thoroughly with an acetic acid solution and deionized water to remove the redundant PVA/CS crosslinking materials, and dried in an oven at 40 °C. Then, the PVA/CS-modified membranes were obtained. The PVA/CS-modified PTFE product was named PTFE-PVA/CS. Thereafter, the as-prepared PTFE-PVA/CS membranes were dipped into the PBA/SiO_2_ aqueous solution for about 20 s. After adsorption for a period of time, the sample was rinsed several times with deionized water and then placed in a vacuum oven at 40 °C. The obtained samples have been named PTFE-PVA/CS-SiO_2_ in this study.

### 2.4 Characterization

The surface morphology and microstructure of the membrane were investigated by field emission scanning electron microscopy (FESEM, Hitachi S-4800, Japan). The surface chemical compositions of the membranes were studied by X-ray photoelectron spectroscopy (XPS, Kratos, XSAM 800, US) and attenuated total reflectance Fourier transform infrared spectroscopy (ATR-FTIR, Nicolet 5700, US). The hydrophilicity of the modified membrane was characterized by a static water contact angle goniometer (WCA, JY82B, Chengde Dingsheng Testing Machine Co. Ltd. China) and water flux. A pore size analyzer (Capillary Flow Porometer, CFP-1500AE, America) was used to study the pore size distribution of the different PTFE flat membranes. The droplet sizes of the oil-in-water emulsion, filtrate and Tween-80 solution were measured by a dynamic light scattering (DLS) laser particle size analyzer (Nano-s, UK). The concentration of the oil-in-water emulsion was determined by a UV-Vis spectrometer (Lambda 900, America).

### 2.5 Hydrophilic property testing of the modified membrane

The hydrophilic properties of the modified membranes are usually characterized by static water contact angles and water flux. Water contact angles were measured on dried membranes by a contact angle goniometer equipped with a video capture device.^33^

A homemade dead-end filtration system was developed for testing the pure water flux. The effective diameter of the membrane was 4.2 cm, and the trans-membrane pressure was 0.05 MPa. Note that the membrane coupons loaded in the filtration cells were pressured at 0.1 MPa using deionized water for at least 1.0 h to ensure a stable membrane flux before testing. The water flux was calculated by eqn (1).1
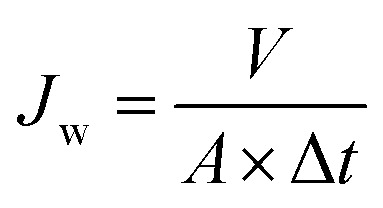
where *J*_w_ is the water permeation flux (L m^−2^ h^−1^), *V* is the volume of permeation (L), Δ*t* is the test time (h) and *A* is the effective area of the membrane (m^2^).

### 2.6 Preparation and separation of the oil-in-water emulsion

The oil-in-water emulsion was prepared by mixing 0.75 g vegetable oil, 997 mL deionized and 0.13 g Tween-80, which was treated by mulser stirring at 10 000 rpm for 75 min. The droplet size of the oil-in-water emulsion was in the range of 68.06–712.38 nm, as detected by dynamic light scattering.

The hydrophilic PTFE membranes were first fixed in a sand-core filter with the inner diameter of 4 cm, and then, the oil–water emulsion was poured into a glass tube at room temperature; the experiment was carried out under the trans-membrane pressure of 0.01 MPa. The permeation flux was calculated using eqn (1). The oil content in the water was measured by a UV-Vis spectrometer (Lambda 900, America) at 280 nm, and the separation efficiency was calculated by the oil rejection efficiency according to eqn (2).^34,35^2
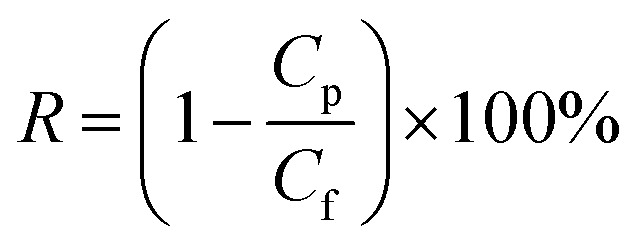
where *R* (%) is the oil rejection efficiency, *C*_f_ is the oil concentration of the oil-in-water emulsion, and *C*_p_ is the oil concentration of the collected water. The antifouling performance was evaluated by a three-cycle filtration method. After each cycle, the oil layer on the surface of the membrane was first rinsed off with ethanol, and then, the membrane was cleaned with deionized water by filtration for 5 minutes.

### 2.7 Tests for the antibacterial properties

For the inhibition zone, *S. aureus* (ATCC strain 29523) and *E. coli* (ATCC strain 29522) were used to evaluate the antibacterial activity of the PTFE, PTFE-PVA, PTFE-PVA/CS and PTFE-PVA/CS-SiO_2_ membranes. The bacterial suspension (1 mL) was taken out by pipette, poured into an aureus agar plate, and coated uniformly by a glass coating rod. Then, different flat PTFE membranes were loaded onto a sterile blank displaced on the surface of the agar. After this, the aureus agar plate was incubated at 37 °C for 24 h. The inhibition zone was determined by a digital camera.

## 3. Results and discussion

**Fig. 2:**
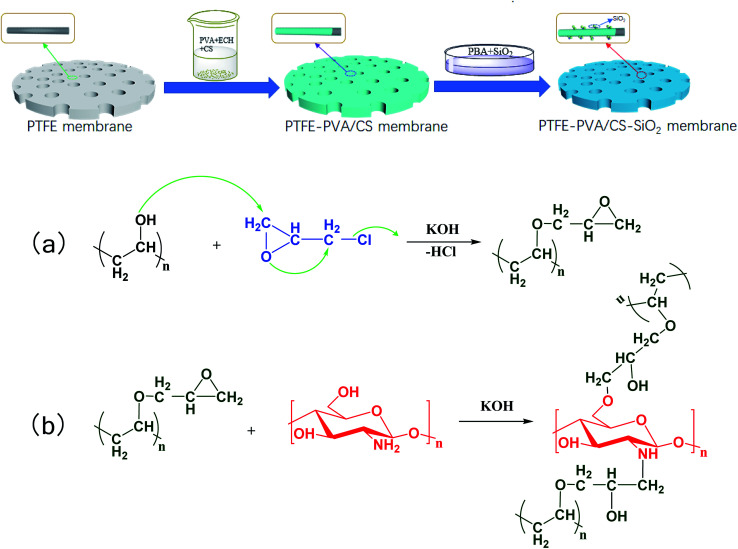
Schematic of the whole fabrication process for the PVA/CS-SiO_2_-modified PTFE membrane and the possible chemical reaction mechanism.

The schematic of the PVA/CS-SiO_2_-modified PTFE membrane and the possible chemical reaction mechanism are shown in Fig. 2. ECH can react with hydroxyl groups or amino groups to form secondary alcohols under alkaline conditions. When ECH was added to a solution containing PVA and CS, PVA and CS were cross-linked *via* ECH to form a three-dimensional network of macromolecules, which was deposited and wrapped on the node and fiber surface to form the PVA/C layer. Using PBA as an adhesive, the SiO_2_ particles were bonded to the PTFE-PVA/CS membrane, which further improved the hydrophilicity of the modified membrane.

### 3.1 The surface chemical structure of membranes

**Fig. 3:**
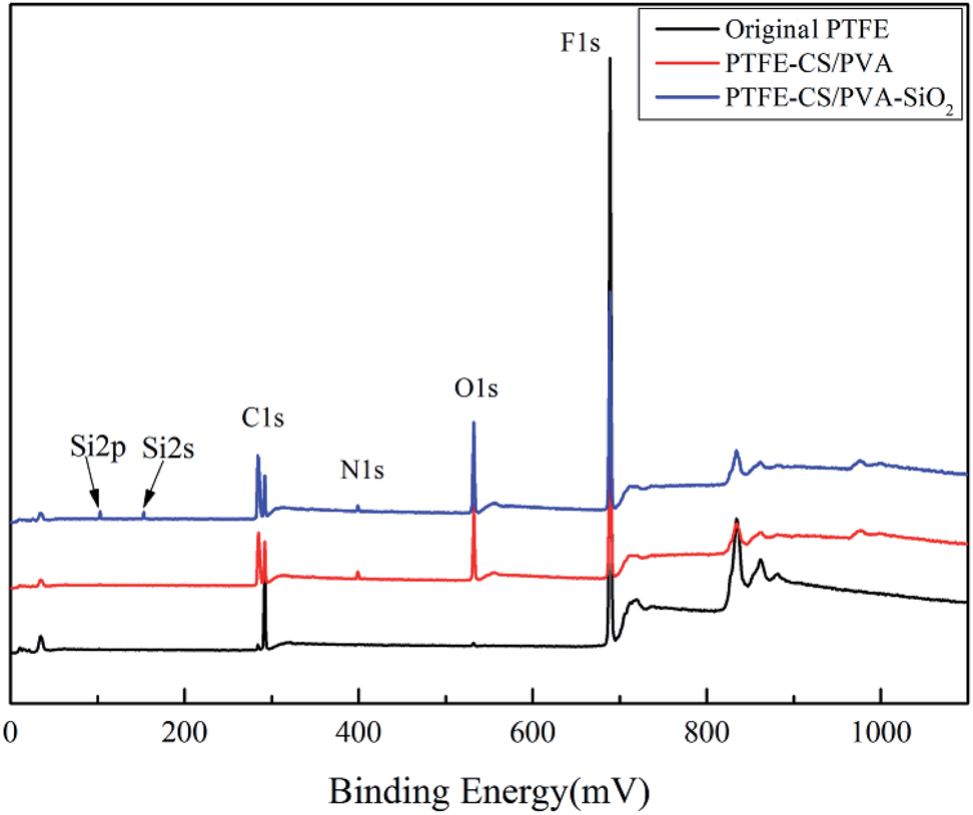
XPS spectra of different PTFE flat membranes.

The elemental compositions of the different PTFE flat membranes were determined *via* XPS; the survey scanning spectra of the membranes are shown in Fig. 3. The original membrane only showed the peaks of C1s and F1s at 284.75 eV and 689.29 eV, respectively. Compared to the case of the original membrane, the characteristic peaks of O1s and N1s were detected for PTFE-PVA/CS, which indicated the occurrence of cross-linking reactions. In addition, the new peaks of Si2s and Si2p originated after modification of the membrane by SiO_2_. It was not difficult to draw a conclusion that the membrane surface had absorbed silica nanoparticles. The results showed that PVA/CS and SiO_2_ were successfully attached to the surface of the PTFE flat membrane. Finally, the elemental composition and content of the different membrane surfaces were determined and are presented in Table 1, corresponding to the results of the XPS spectra.

**Table 1:** Elemental compositions of different PTFE flat membranes

Membrane	Composition (at%)				
	C	F	O	N	Si
Original PTFE	33.17	66.83	—	—	—
PTFE-PVA/CS	38.42	41.75	19.12	0.72	—
PTFE-PVA/CS-SiO_2_	40.63	37.93	20.02	0.48	0.95

**Fig. 4:**
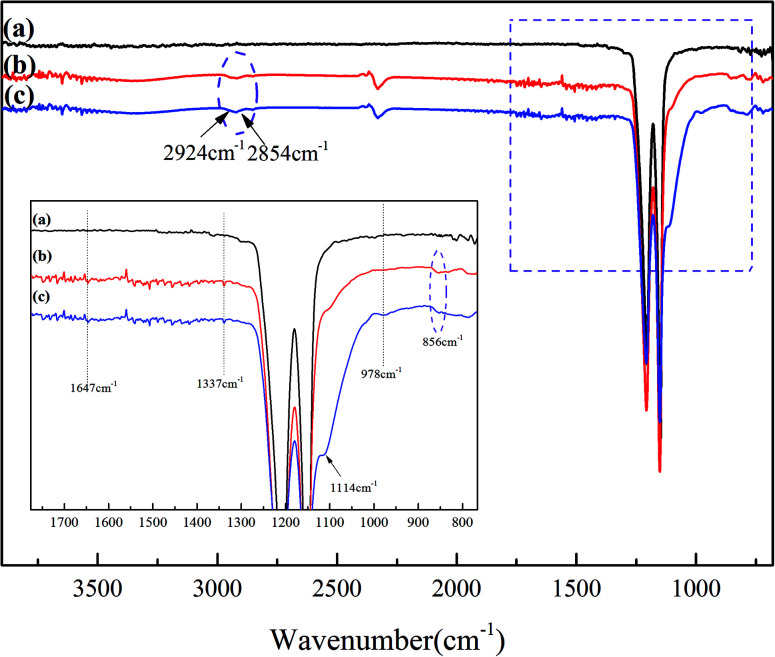
ATR-FTIR spectra of different membrane surfaces: original PTFE (a), PTFE-PVA/CS (b) and PTFE-PVA/CS-SiO_2_ (c) flat membranes.

The chemical groups of the membranes were characterized by ATR-FTIR. Fig. 4 shows the ATR-FTIR spectra of the original PTFE membrane, the PTFE-PVA/CS membrane and the PTFE-PVA/CS-SiO_2_ membrane. The two peaks obtained at 1149 and 1205 cm^−1^ can be related to the asymmetric stretching of the –CF_2_ groups on the original and modified PTFE membrane surface, respectively. As shown in Fig. 4b, compared with the case of the pristine membrane, new absorption peaks appeared in the curve of PTFE-PVA/CS. The absorption peaks in the range of 3700–3000 cm^−1^ are ascribed to the stretching vibrations of –OH and can overlap with the N–H bands of amine and amide. The absorption peaks at 2924 cm^−1^ and 2854 cm^−1^ were ascribed to the symmetric stretching of the –CH_2_– bond. The bands at 1647 cm^−1^ and 1337 cm^−1^ are due to the presence of –NH_2_ bending vibrations and C–H symmetric bending vibrations in –CHOH, respectively. In addition, the modified membrane surface shows two new peaks at 978 cm^−1^ and 856 cm^−1^, which are assigned to –C–O and glycosidic C–O–C stretching vibrations, respectively.^36,37^ Moreover, after soaking the membrane in a silicon sphere solution, a new peak was obtained at 1114 cm^−1^, demonstrating the existence of nano-SiO_2_; in summary, the FTIR spectroscopy results demonstrated the successful synthesis of PTFE-PVA/CS-SiO_2_; moreover, these results correspond to the XPS results.

### 3.2 The surface morphological structure of membranes

**Fig. 5:**
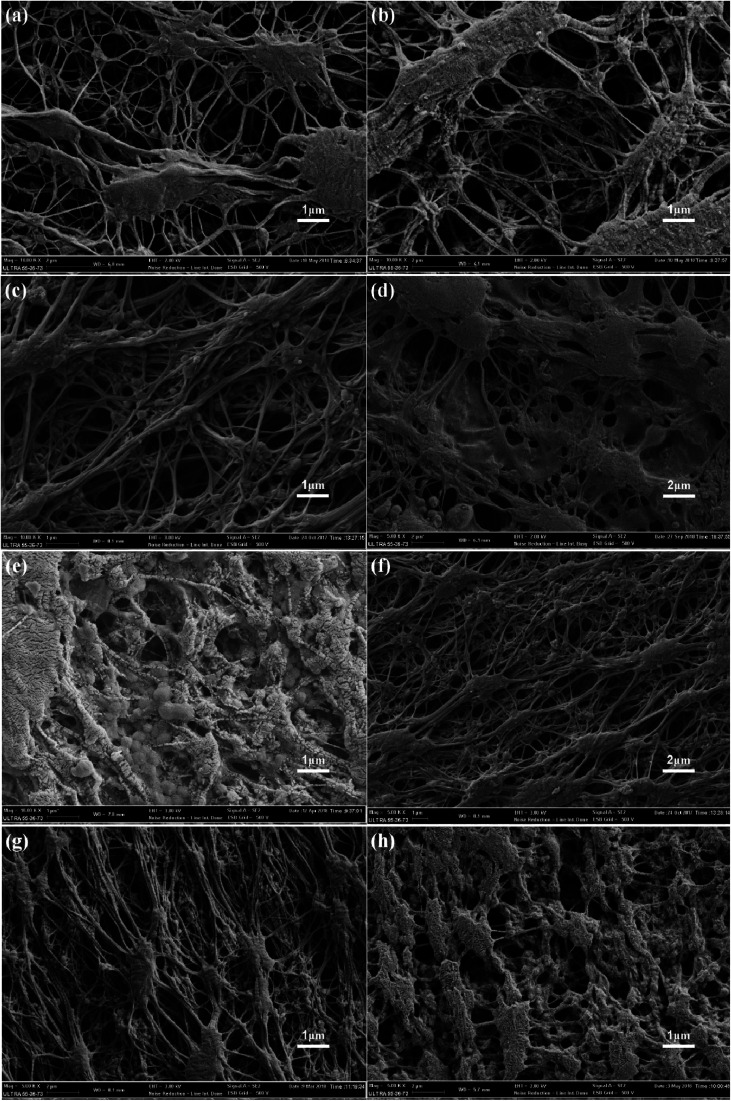
SEM images of different membrane surfaces. Original PTFE (a), PVA/CS solution treatment (no ECH) (b), PTFE-PVA/CS (1 wt% PVA and 0.3 wt% CS mass ratios of 1.5 : 1 and 0.5 : 1) (c and e), PTFE-PVA/CS (PVA content of 5 wt%) (d), PTFE-PVA/CS-SiO_2_ (DF is 50 times, 45 times and 25 times) (f–h).

The surface morphology changes of the membranes were determined using FESEM, as shown in Fig. 5. The surface morphologies of the original membranes are shown in Fig. 5a, and it can be found that these membranes are composed of nodes and fibrils. As shown in Fig. 5b, there were no obvious changes after the pre-reaction solution treatment; this indicated that the uncrosslinked PVA or CS could not modify the PTFE membrane. The surface morphology of the membrane obtained under the optimal reaction conditions, as shown in Fig. 5c, indicates that the fibrils of the membrane are covered with a new layer of material, and no blockage occurs. As shown in Fig. 5d and e, the micro-pores of the membrane were blocked when there was high concentration of PVA or CS. Compared to the case of the PVA/CS-modified membrane, coagulant silicon particles were found on the surface of the PTFE-PVA/CS-SiO_2_ membrane (Fig. 5f–h). The content of SiO_2_ increased with a decrease in DF; however, since the agglomeration was highly severe as the DF was reduced from 45 times (Fig. 5g) to 25 times (Fig. 5h), the membrane pores were severely blocked.

**Fig. 6:**
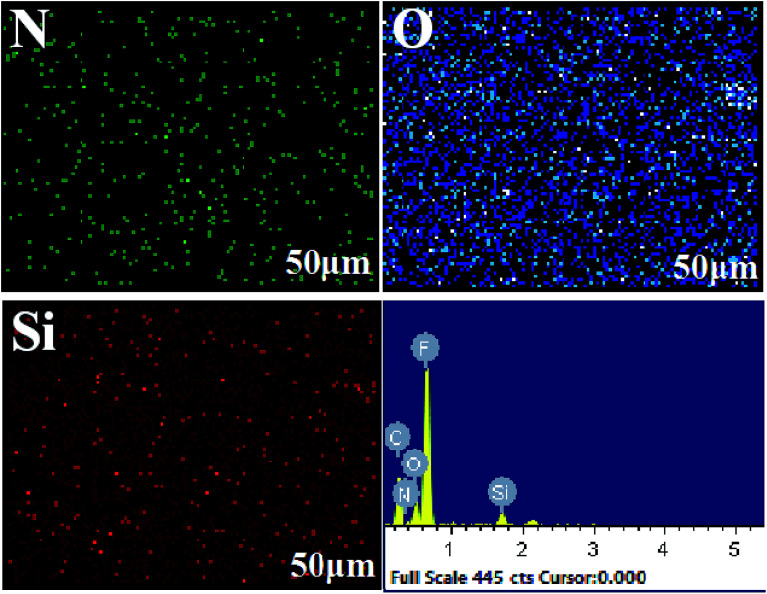
EDX spectra of the PTFE-PVA/CS-SiO_2_ cross-section.

The cross-section EDX scan images of the PTFE-PVA/CS-SiO_2_ membrane are shown in Fig. 6. It can be found that the N, O, Si elements are uniformly distributed inside the pores of the PTFE-PVA/CS-SiO_2_ membrane; this indicates that the entire membrane has been completely modified by PVA/CS-SiO_2_.

### 3.3 Hydrophilicity of the membranes

To evaluate the hydrophilic capacity of the modified membrane, water flux (*J*_w_) was used to evaluate the water permeability, and water contact angle (WCA) was used to evaluate the wettability.

**Fig. 7:**
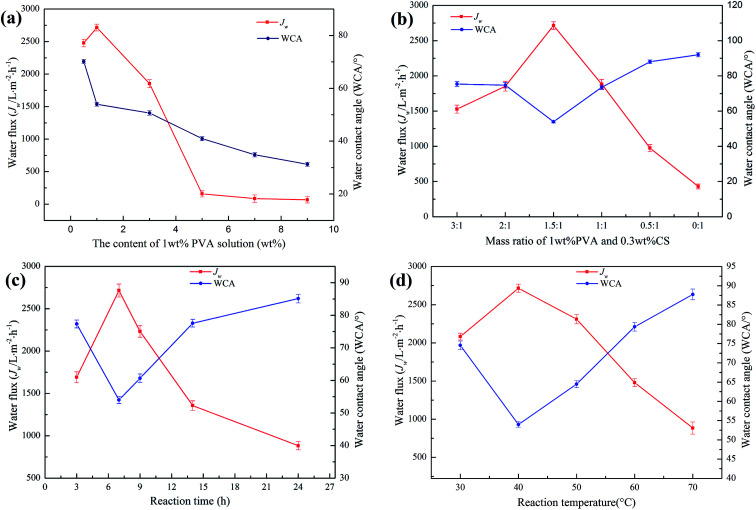
The effects of reaction conditions on the water flux and water contact angle of the membrane (permeation pressure of 0.05 MPa). The content of PVA solution (a); mass ratio of 1 wt% PVA and 0.3 wt% CS (b); and reaction time (c) and temperature (d).

Fig. 7a shows the effect of the PVA content on the hydrophilic properties of the PTFE-PVA/CS membrane. The maximum *J*_w_ was achieved as 2715.19 ± 53 L m^−2^ h^−1^ when the PVA content was 1 wt%; when the PVA content was less than 1 wt%, the *J*_w_ increased with an increase in the PVA content. However, the high PVA content of 9 wt% resulted in a decrease in *J*_w_, which was around 67.74 L m^−2^ h^−1^. When the content of PVA was very high, a large amount of PVA did not react. After the adhesion of some PVA, the remaining PVA adhered to the surface of the modified membrane, causing the pores of the membrane to clog and the water flux to decrease. Moreover, the WCA continued to decrease due to an increase in the number of hydrophilic groups on the surface of the membrane. Fig. 7b shows the effect of the mass ratio of 1 wt% PVA and 0.3 wt% CS on the hydrophilic properties of the PTFE-PVA/CS membrane. The best *J*_w_ and WCA were 2715.19 ± 53 L m^−2^ h^−1^ and 53.98 ± 0.7°, respectively, when the mass ratio was 1.5 : 1. As the CS content decreases, the probability of the reaction of ECH with CS increases, and the insufficient hydrophilicity of CS results in a decrease in *J*_w_ and an increase in WCA. The effect of reaction time on the hydrophilicity of the modified membrane was investigated (Fig. 7c). The best reaction time was 7 h; when the reaction time was too long, it resulted in an increase in the degree of cross-linking, and the number of hydrophilic groups decreased; therefore, the hydrophilicity decreased. Reaction temperature also plays an important role in membrane hydrophilization (Fig. 7d). When the reaction temperature is too high, the cross-linking speed is increased, causing the ECH to cross-link PVA and CS in the solution; hence, less ECH enters the pores of the membrane, and the cross-linked hydrophilic layer is less. Thus, the best reaction temperature is 40 °C.

Based on the abovementioned findings, when there is a large number of hydrophilic layers on the surface of the membrane and there is no blockage of the membrane pore, the optimal reaction conditions are as follows: the content of the PVA solution, mass ratio of PVA and CS, reaction time and temperature are 1 wt%, 1.5 : 1, 7 h and 40 °C, respectively. Therefore, the PTFE-PVA/CS membrane was prepared under these optimal reaction conditions for the subsequent experiment.

**Fig. 8:**
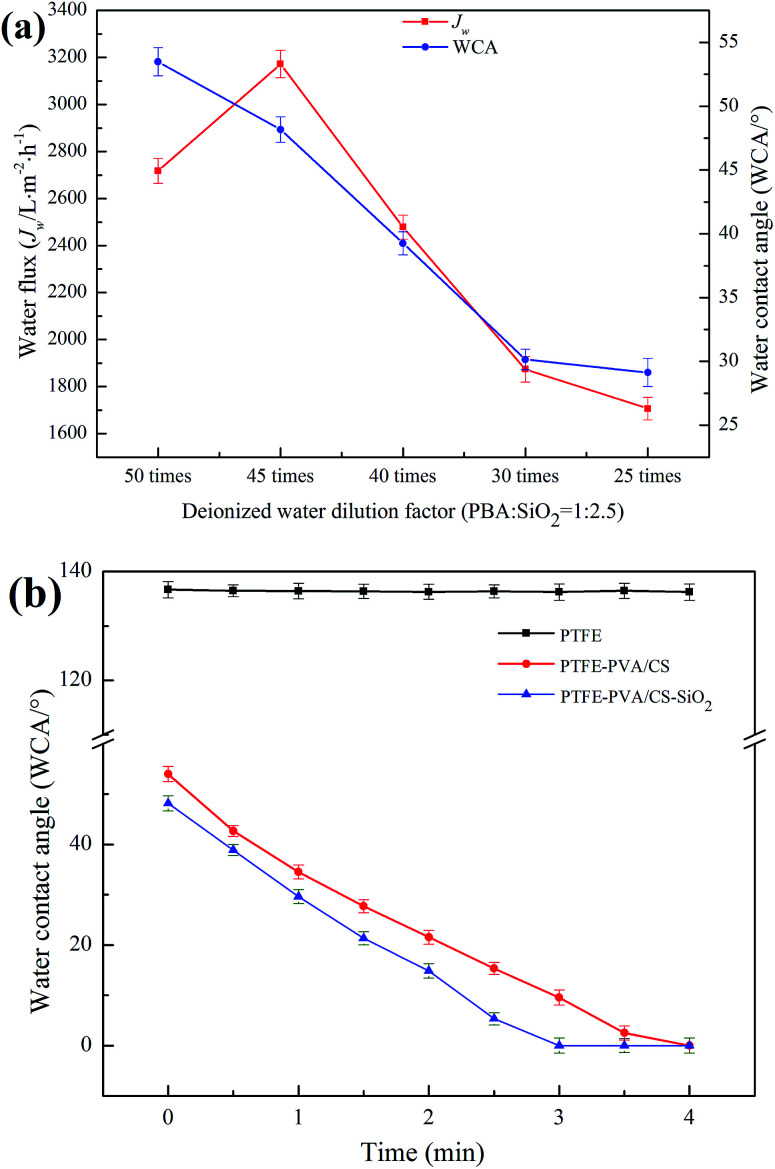
The effects of PBA/SiO_2_ content on the hydrophilicity of the modified PTFE flat membrane (a) and water contact angle of different membranes *versus* time (b).

After the PBS/SiO_2_ treatment, the hydrophilicity of the membrane was further improved due to the strong hydrophilicity of SiO_2_. The effect of deionized water dilution factor (DF) of PBA/SiO_2_ on the hydrophilicity of the PTFE-PVA/CS membranes is shown in Fig. 8a. The WCA decreased from 53.48° ± 1.1° to 29.13° ± 1.1° as the DF of PBA/SiO_2_ decreased from 50 times to 25 times; when the DF was 45 times, the *J*_w_ of the modified membrane reached maximum, which was 3171.91 ± 58 L m^−2^ h^−1^. However, high or low DF caused a decrease in the *J*_w_ of the PTFE-PVA/CS-SiO_2_ membrane. When the DF was too high, the SiO_2_ concentration was low, and there was less adhesion on the surface of the modified membrane; thus, the hydrophilicity of the membrane was poor. When the DF was too low, the SiO_2_ concentration was too high, causing the pores of the membrane to be blocked; this resulted in a decrease in water flux; however, the surface hydrophilic groups increased; thus, the water contact angle decreased. The dynamic WCA tests (Fig. 8b) also support the abovementioned result. The PTFE-PVA/CS-SiO_2_ membranes show better water permeation rates than the PTFE-PVA/CS membranes; the former requires about 3 min to be completely wetted in air, whereas the latter requires about 4 min.

Since the PTFE-PVA/CS-SiO_2_ (45 times) membrane showed the best water permeability and wettability, all the subsequent experiments were conducted on this membrane.

### 3.4 Oil-in-water emulsion separation

**Fig. 9:**
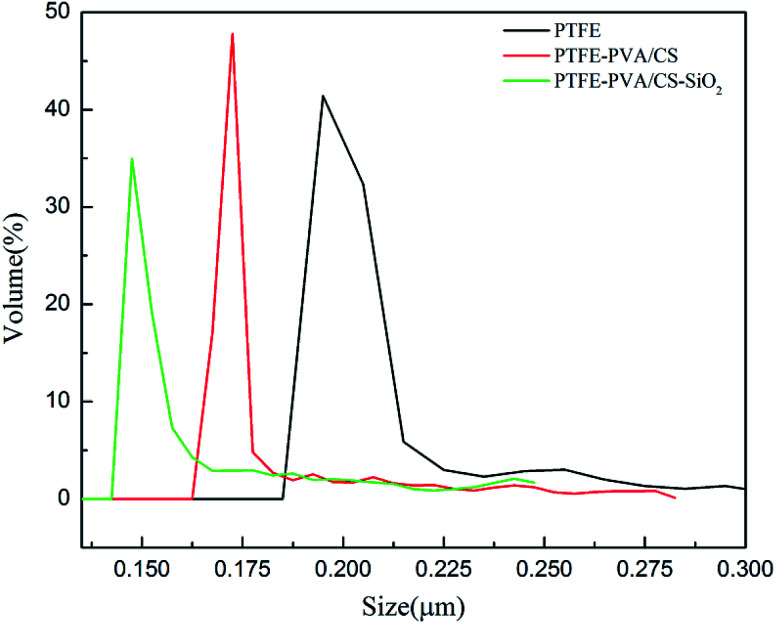
The pore size distribution images of different PTFE membranes.

The pore size of a membrane plays an important role in oil–water separation. The pore size distributions of the original PTFE, PTFE-PVA/CS and PTFE-PVA/CS-SiO_2_ membranes are shown in Fig. 9, and it can be found that the pore size decreases after modification of the membranes by CS or CS-SiO_2_. The mean pore size of the original PTFE, PTFE-PVA/CS and PTFE-PVA/CS-SiO_2_ membrane is 0.22 μm, 0.186 μm and 0.164 μm, respectively.

**Fig. 10:**
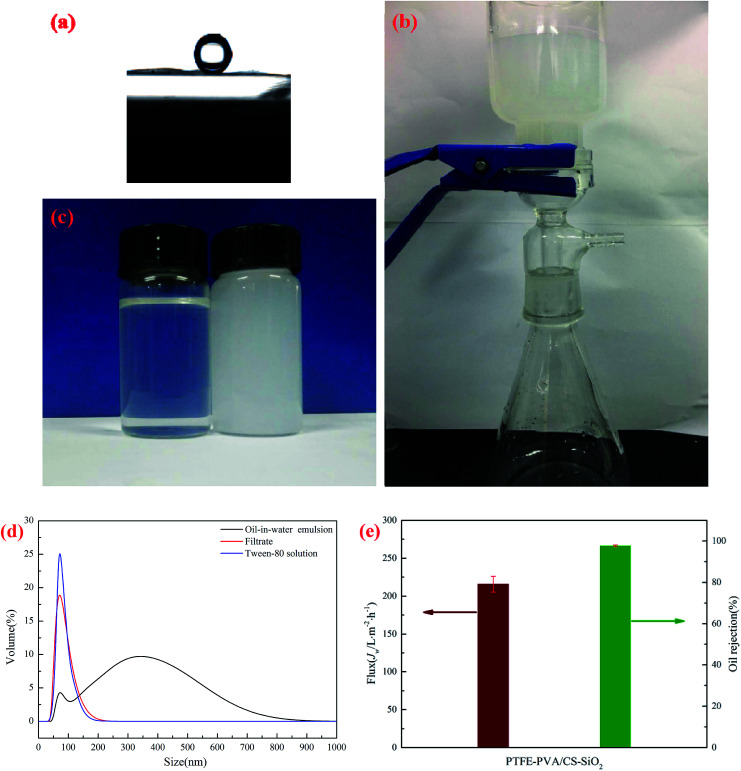
Underwater oil contact angles on the surfaces of the PTFE-PVA/CS-SiO_2_ membrane (a). Images of a vacuum suction filter device (b), oil-in-water emulsion (right) and filtrate (left) (c). DLS data of the oil-in-water emulsion, filtrate and Tween-80 solution (d). Flux and oil rejection of the PTFE-PVA/CS-SiO_2_ membrane (e).

The underwater oil contact angle and antifouling performance of the PTFE-PVA/CS-SiO_2_ membrane were investigated. As shown in Fig. 10a, the underwater contact angle of the PTFE-PVA/CS-SiO_2_ membrane is 158.47° ± 1.0°, indicating that the modified membrane has super-oleophobic properties under water and can be used to separate oil-in-water emulsions.

The separation performance of the PTFE-PVA/CS-SiO_2_ membrane for oil-in-water emulsions was studied in detail. A vacuum suction filter device was used under the pressure of 0.01 MPa (Fig. 10b). The separation results for oil-in-water emulsions are shown in Fig. 10c; the color of the oil-in-water emulsion is milky, and the filtrate becomes clear after filtration. As shown in Fig. 10d, the droplet size of the emulsion is in the range of 50.75–824.99 nm, and a sharp peak appears in the range of 43.82–220.19 nm of the filtrate; this peak can be attributed to the residual Tween-80 in the filtrate.^38^ To further confirm this point, a control experiment was conducted. In the control experiment, 0.13 g Tween-80 was dissolved in 1000 mL of water, and as expected, a similar peak was observed around 37.84–220.19 nm, confirming that this sharp peak was caused by the residual Tween-80 in the filtrate. The results showed that most of the oil in the water was successfully removed from the oil–water emulsion. The corresponding calculations were performed, and the permeate flux and rejection of the oil-in-water emulsion under 0.01 MPa transmembrane pressure are shown in Fig. 10e. The oil rejection of the PTFE-PVA/CS-SiO_2_ towards oil-in-water emulsions is above 97.67 ± 0.5%, whereas the filtrate flux is 215.76 ± 10.3 L m^−2^ h^−1^.

**Fig. 11:**
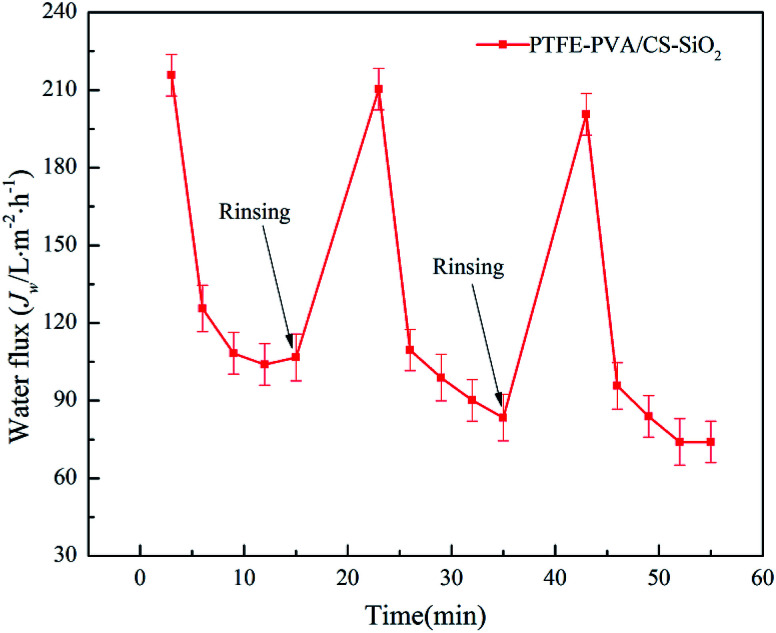
PTFE-PVA/CS-SiO_2_ membrane flux recovery over three cycles.

The antifouling property of the membrane is critical during the oil–water separation process. The oil-in-water emulsion filtration experiments were performed for three cycles, and only simple rinsing with water was performed prior to each filtration. The permeation flux value of each filtration is shown in Fig. 11. It can be seen that as the filtration time increases, the permeation flux decreases sharply and tends to be stable. This is because when the membrane contacts the oil–water emulsion, the water and surface hydrophilic substance of the membrane form a strong “aqueous layer”, thereby separating the oil and water. However, as the filtration time increases, the water will form an oily layer on the surface of the membrane, leading to reduced permeation flux. However, after washing the membrane with ethanol and water, the initial permeation flux of the membrane was substantially recovered. These results indicate that both PTFE-PVA/CS-SiO_2_ membranes possess excellent anti-fouling performance and long-term usage.

Compared with the membranes used for oil/water emulsion separation in recent studies (Table 2), the PTFE-PVA/CS-SiO_2_ membrane used herein allowed the separation to be operated under low pressure, and the flux under unit pressure was higher as compared to the case of most existing membranes. Although there is still much work to be implemented, the PTFE-PVA/CS-SiO_2_ membranes have shown their superior performances for efficient oil/water emulsion separation.

**Table 2:** Comparison of the membrane properties for oil/water emulsion separation with other works

Membrane	Operation method	Flux (L m^−2^ h^−1^ bar^−1^)	Pressure (bar)	Reject (%)	Reference
PTFE-PVA/CS-SiO_2_	Dead end	2157.6	0.1	97.67	This work
PVDF@pDA@SiO_2_	Dead end	572	0.8	98	40
APTES@PVDF/GO	Dead end	1000	0.5	99.8	39
UiO-66-NH_2_(1)@PAA	Dead end	2330	0.1	99.9	42
PVDF/DA/TiO_2_ 12%	Dead end	573	0.3	98.6	41
WO_3_/TiO_2_	Dead end	1300	1	98.5	44
PVDF/TPTi	Dead end	556.6	0.5	99.9	43

### 3.5 Antibacterial property

**Fig. 12:**
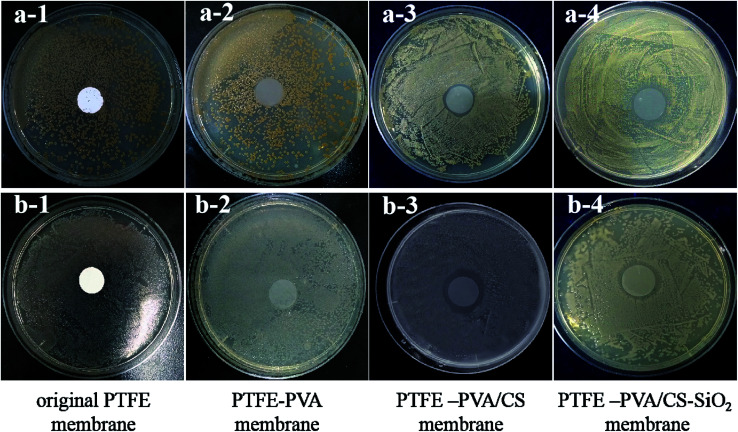
Antibacterial properties of the original PTFE, PTFE-PVA, PTFE-PVA/CS and PTFE-PVA/CS-SiO_2_ membrane on *S. aureus* culture (Gram-positive bacteria) (a) and *E. coli* culture (Gram-negative bacteria) (b).

The antibacterial activities of the original PTFE, PTFE-PVA, PTFE-PVA/CS flat membranes were investigated against the Gram-positive *S. aureus* culture and the Gram-negative *E. coli* culture by the zone of inhibition tests. The results are shown in Fig. 12, and it can be seen that colonies are still present below the PTFE-PVA membrane, whereas the colonies below the PTFE-PVA/CS membranes completely disappear, and there is a zone of inhibition. The diameters of the zones of inhibition for the *S. aureus* and *E. coli* cultures were 1.5 mm and 2 mm, respectively. It can be seen that the antibacterial performance of the membrane against *E. coli* is greater than that against *S. aureus*; this indicates that this membrane has certain selectivity. In the PTFE-PVA/CS-SiO_2_ membrane, since the SiO_2_ layer is not a dense layer, CS can still act as an antibacterial agent; therefore the modified membrane has antibacterial property.

### 3.6 Stability test of the modified membranes

The stability of the hydrophilic properties of the modified membrane is critical to the membrane. The hydrophilic stability, including physical and chemical stability, of the modified membrane is usually characterized by the treated water flux and contact angle.

**Fig. 13:**
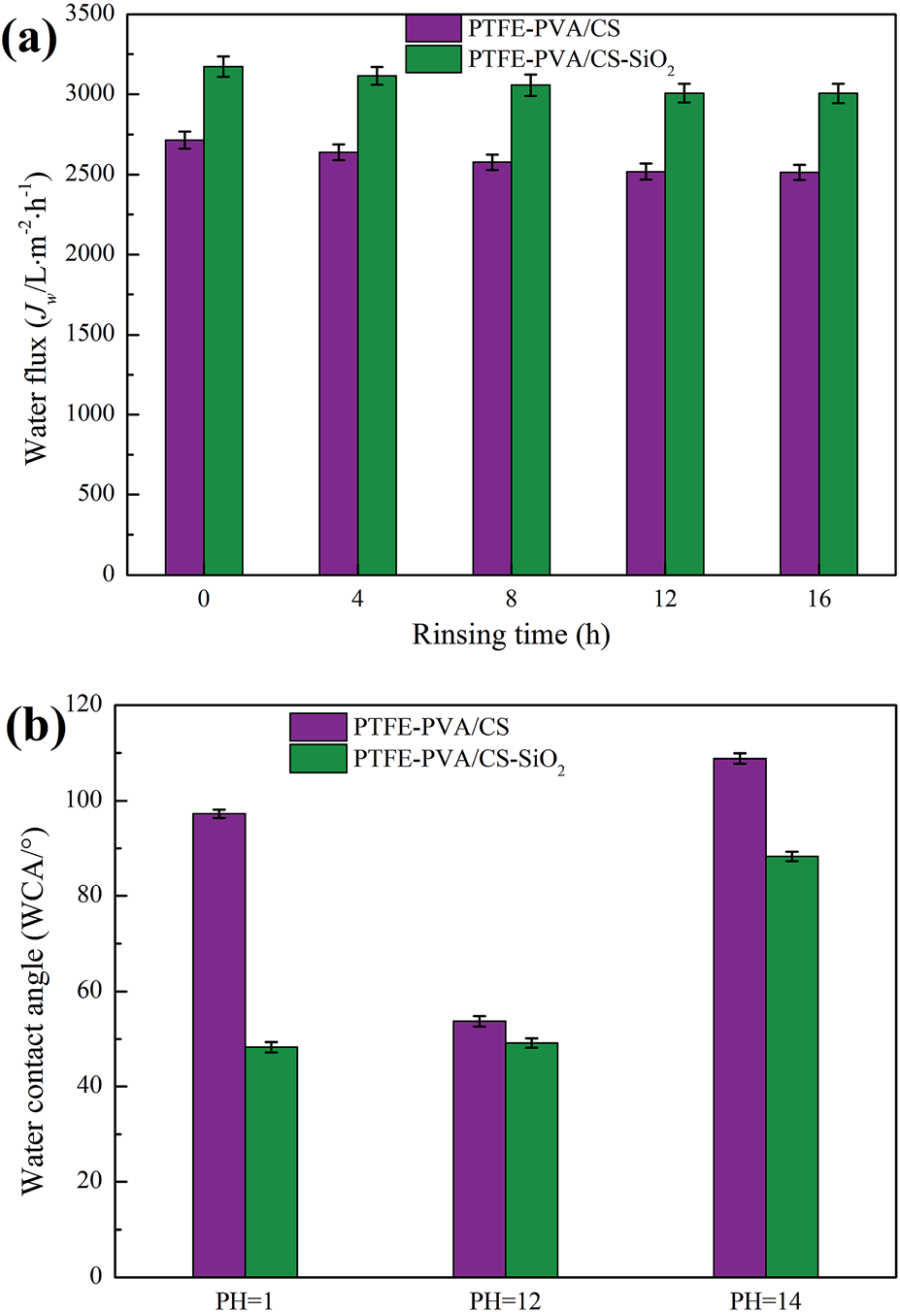
The stability of modified membranes with different solution treatments: physical stability (a) and chemical stability (b).

To characterize the physical stability of the PTFE-PVA/CS and PTFE-PVA/CS-SiO_2_ membranes, the modified membranes were continuously rinsed with deionized water for 16 hours in this study, and the *J*_w_ values of the modified membranes were tested every 4 hours. As shown in Fig. 13a, the *J*_w_ of the modified membrane first decreases slightly with an increase in the rinsing time and then tends to be stable. The *J*_w_ loss of the modified membrane is small, indicating that the hydrophilic coating of the modified membrane has good physical stability.

The chemical stability of the modified membrane is crucial in practical applications, especially the acid and alkali resistance of the membrane. To further study the acid and alkali resistance of the modified membrane, the surface WCA of the modified membranes was measured after immersing the membranes in different pH solutions for 12 h in this study, and the results are shown in Fig. 13b. It can be seen that the PTFE-PVA/CS membrane is stable under weakly alkaline conditions; however, the surface WCA of the modified membrane is increased under strongly acidic and alkaline conditions because the hydrophilic coating of the modified membrane is destroyed under these conditions. In contrast, the PTFE-PVA/CS-SiO_2_ membrane exhibits excellent stability under strongly acidic conditions, and the WCA does not significantly change. The main reason is that the SiO_2_ layer prevents the acid from contacting the PVA/CS layer and protects the modified layer from strong acidic conditions. Under the alkaline conditions, the SiO_2_ layer showed the same results as the PVA/CS layer. Moreover, it was stable under weakly alkaline conditions; however, it could not tolerate a strongly alkaline environment.

## 4. Conclusion

In summary, a new hydrophilic and antibacterial membrane was prepared *via* a novel and simple modification method of crosslinking chitosan with polyvinyl alcohol using epichlorohydrin as a cross-linker followed by *in situ* chimeric SiO_2_ nanoparticle adhesion. The modified membrane demonstrated excellent hydrophilicity, anti-bacterial activity and outstanding antifouling performance. In contrast, the PTFE-PVA/CS-SiO_2_ membrane showed better water permeation performance and anti-fouling ability. The modified membrane showed good long-term durability in aqueous environments. Moreover, PVA and CS are non-toxic, cheap, and easily degradable eco-friendly materials. Therefore, this method is promising for practical application.

## Conflicts of interest

There are no conflicts to declare.

## Supplementary Material
